# Annotation

**Published:** 1952-07

**Authors:** 


### *Annotation*

### THE TEACHING OF ANAESTHESIA IN EUROPE

As soon as the World Health Organization had been set up after the war, as an offshoot of the United Nations Organization, requests came from many countries in Europe for help in the development of anaesthesia. While it would have been possible for W.H.O. to grant Fellowships for European practitioners to study in Britain, where it was recognized that this subject was well advanced, the Organization's policy is to assist member countries by bringing experts to them to help in forming centres which can continue to develop later without assistance.

Accordingly, in 1950, an anaesthesia centre for Europe was set up in Copenhagen by the World Health Organization acting with the Danish Government. W.H.O. provided most of the teaching staff and the necessary equipment, and the Danish Government provided the accommodation and the clinical material. As a result of experience gained in the first year it was decided to co-ordinate the teaching under a Chief Instructor from Britain for a one-year course. Dr. R. Woolmer, Bristol University Lecturer in Anaesthesia, appointed to fill this post, has just returned to Bristol on its completion. For part of the course he was assisted by Dr. V. T. Baxter of Bristol United Hospitals. A number of practitioners from ten different countries in Europe have been given a strenuous course of practical and theoretical instruction, which is intended to put them on the right road to specialist rank. The W.H.O. has arranged for some of the more promising students to study for another six months at one or other of the teaching centres in Britain before returning to their own countries.

